# Deciphering the diet of a wandering spider (*Phoneutria boliviensis*; Araneae: Ctenidae) by DNA metabarcoding of gut contents

**DOI:** 10.1002/ece3.7320

**Published:** 2021-03-06

**Authors:** Diego Sierra Ramírez, Giovany Guevara, Lida Marcela Franco Pérez, Arie van der Meijden, Julio César González‐Gómez, Juan Carlos Valenzuela‐Rojas, Carlos Fernando Prada Quiroga

**Affiliations:** ^1^ Grupo de Investigación Biología y Ecología de Artrópodos (BEA) Corporación Huiltur Neiva, Facultad de Ciencias Universidad del Tolima Ibagué Colombia; ^2^ Grupo de Investigación en Zoología (GIZ) Facultad de Ciencias Universidad del Tolima Ibagué Colombia; ^3^ Facultad de Ciencias Naturales y Matemáticas Universidad de Ibagué Ibagué Colombia; ^4^ CIBIO Research Centre in Biodiversity and Genetic Resources InBIO Universidade do Porto Vairão Vila do Conde Portugal; ^5^ Programa de Licenciatura en Ciencias Naturales y Educación Ambiental Facultad de Educación Universidad Surcolombiana Neiva Colombia

**Keywords:** diet, gut content metabarcoding, molecular diet analysis, *Phoneutria*, predator–prey interaction, prey detection

## Abstract

Arachnids are the most abundant land predators. Despite the importance of their functional roles as predators and the necessity to understand their diet for conservation, the trophic ecology of many arachnid species has not been sufficiently studied. In the case of the wandering spider, *Phoneutria boliviensis* F. O. Pickard‐Cambridge, 1897, only field and laboratory observational studies on their diet exist. By using a DNA metabarcoding approach, we compared the prey found in the gut content of males and females from three distant Colombian populations of *P. boliviensis*. By DNA metabarcoding of the cytochrome *c* oxidase subunit I (COI), we detected and identified 234 prey items (individual captured by the spider) belonging to 96 operational taxonomic units (OTUs), as prey for this wandering predator. Our results broaden the known diet of *P. boliviensis* with at least 75 prey taxa not previously registered in fieldwork or laboratory experimental trials. These results suggest that *P. boliviensis* feeds predominantly on invertebrates (Diptera, Lepidoptera, Coleoptera, and Orthoptera) and opportunistically on small squamates. Intersex and interpopulation differences were also observed. Assuming that prey preference does not vary between populations, these differences are likely associated with a higher local prey availability. Finally, we suggest that DNA metabarcoding can be used for evaluating subtle differences in the diet of distinct populations of *P. boliviensis*, particularly when predation records in the field cannot be established or quantified using direct observation.

## Graphical abstract

Arachnids are the most abundant land predators. Despite the importance of their functional roles as predators and the of necessity to understand their diet for conservation and nutrient fluxes, there is a small amount of work on trophic ecology in this group of arachnids. By using a DNA metabarcoding approach, we compared the prey found in the gut content of males and females from three distant Colombian populations of *P. boliviensis*. These results suggest that *P. boliviensis* feeds predominantly on invertebrates (Diptera, Lepidoptera, Coleoptera, and Orthoptera) and opportunistically on small squamates. Intersex and interpopulation differences are observed. Assuming that prey preference does not vary between populations, these differences are likely associated with a higher local prey availability.

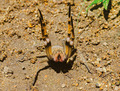

## 1 INTRODUCTION

Understanding the contribution of predators in shaping the structure of ecological communities is a central issue in ecology (Lima, 1998; Schmitz, 2007; Seibold et al., 2018). Predator–prey interactions are a main driver of natural selection, population dynamics, food web structure, community assembly, and ecosystem functioning (Portalier et al., 2019; Severtsov & Shubkina, 2015; Start et al., 2020). Spiders are among the most abundant predators in terrestrial ecosystems, playing an important role in controlling prey species populations (Betz & Tscharntke, 2017; Michalko et al., 2019; Pekár et al., 2011; Pusceddu et al., 2018). Almost all spiders are carnivores, feeding predominantly on arthropods including, to a lesser extent, other spiders (Klaus Birkhofer & Wolters, 2012; Nyffeler, 1999; Pekár & Toft, 2015). Very rarely nonarthropod prey are consumed as a supplement to the arthropod diet (Foelix, 2011; Nyffeler et al., 2016; Symondson et al., 2002). However, this appears to be relatively frequent in some spider families which include large‐sized species, such as Theraphosidae, Ctenidae, Lycosidae, and Pisauridae, among others (Hazzi, 2014; Valdez, 2020).

Predators in their search for food sources can develop narrow (stenophagous) or broad (euryphagous) eating habits (Pekár & Toft, 2015). In the case of spiders, the stenophagous specialists possess adaptations for the capture of large focal prey, minimizing handling time, and live in proximity to their prey so they can minimize foraging time. As a result, the capture time is much shorter than in euryphagous species (García et al., 2018; Michálek et al., 2017; Pekár et al., 2011; Pompozzi et al., 2019). On the other hand, euryphagous spiders, which select prey smaller than their body and must thus capture more items of prey, could minimize foraging time by shortening the duration of consumption of each item of prey (García et al., 2016; Pompozzi et al., 2019). Indeed, recent studies showed that cursorial obligatory stenophagous species selected larger prey and fed for a significantly longer time, extracting significantly more mass than euryphagous spiders (García et al., 2018; Michálek et al., 2017).

Spiders of the genus *Phoneutria*, popularly known as “Banana spiders” or “wandering spiders” are restricted to South America and they are essentially wandering, nocturnal spiders. This genus represents one of the main groups of medically important spiders in South America because of their defensive behavior, synanthropic habits, and potent venom (Hazzi, 2014; Valenzuela‐Rojas et al., 2019; Vetter & Isbister, 2008). Recent records on the diet of *P. boliviensis* suggest these spiders prey on several arthropod species but also consume vertebrates, mainly reptiles and anurans (Valenzuela‐Rojas et al., 2019). In addition, mammals and birds have occasionally been reported as prey in other *Phoneutria* species, suggesting they are likely a generally euryphagous genus (Bücherl et al., 2013).

Intersexual differences have been reported in the production of venom of *Phoneutria*. For example, experiments with *P. nigriventer* and *P. boliviensis* showed that there is greater venom production in females than in males; in *P. boliviensis* females, the venom quantity released was almost three times that in males (Estrada‐Gomez et al., 2015; Herzig et al., 2002; Valenzuela‐Rojas et al., 2019), which could indicate that the amount of venom produced may reflect differences in prey preference because a larger amount may be needed to immobilize larger or more dangerous prey. However, recent laboratory observations on *P. boliviensis* showed no difference in prey acceptance between males and females (Valenzuela‐Rojas et al., 2019). In this same study, the venom was found to be more effective against vertebrate (geckos) and spider prey than other prey types, suggesting that the quantity of venom production could be directly related to prey choice or serve a defensive function for Phoneutria.

Spiders feed on the predigested fluids of their prey through external digestion and ingest nutrients only in liquid form. Following ingestion of liquefied material through the esophagus and sucking stomach, the food enters the midgut which branches into highly complex diverticula extending throughout the b a, opisthosoma and even into the legs (Foelix, 2011; Macías‐Hernández et al., 2018). Consequently, digestion takes place in many different parts of the body and dissecting the whole gut is challenging due to its complexity and extent (Macías‐Hernández et al., 2018). This condition thus restricts studies of spider diet (Birkhofer et al., 2017; Jackson et al., 2001; Lafage et al., 2020; Pompanon et al., 2012). Diet studies involving the taxonomic determination of prey items by field observation depend largely on the experience of the researcher, and are therefore not only labor‐intensive, but potentially biased.

Molecular gut content analysis is a valuable tool for characterizing trophic interactions where conventional diet records are difficult to establish. For the analysis of spider diet, DNA‐based methods thus possess a range of advantages over the classical approaches in diet analyses (Lafage et al., 2020; Sheppard & Harwood, 2005). DNA metabarcoding has proven to be a very accurate and efficient tool for the analysis of spider diets, capable of complementing other techniques for the detection of prey, and detecting previously unnoticed prey in ecological studies on multiple species of spiders with euryphagous diets (Lafage et al., 2020; Piñol et al., 2019).

For the first time, we deploy a DNA metabarcoding approach to study the diet of *Phoneutria boliviensis*, a widely distributed spider species in South America. The main objective of this study is to establish the breadth of the diet of *P. boliviensis*. Furthermore, we will test whether there are interpopulation differences in diet. Since this species is known to have sexual differences in venom production (Valenzuela‐Rojas et al., 2019), which may reflect a difference in prey handling capacity, we also investigate if any differences in diet exist between the sexes.

## 2 MATERIALS AND METHODS

### 2.1 Collection and locations

**FIGURE 1:**
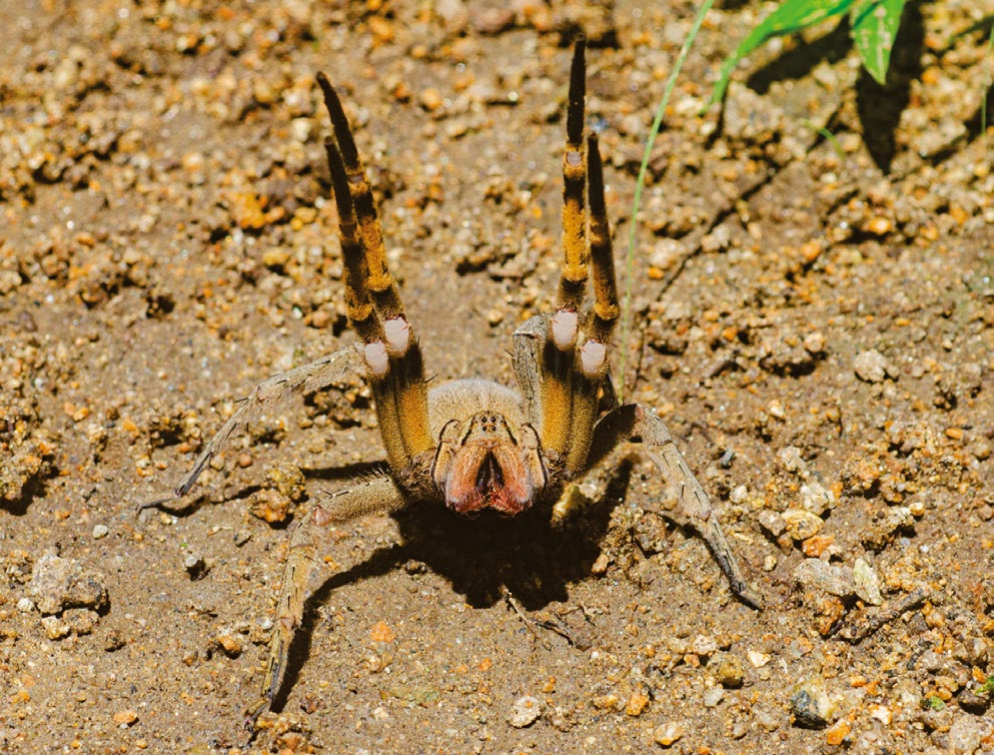
Photo of *Phoneutria boliviensis* in nature

Sixty adult specimens of *P. boliviensis* were used for DNA metabarcoding of the entire gut contents (Figure 1). From each of three Colombian localities, we used twenty individuals (ten females and ten males per locality). These localities are separated by approximately 300 km: Barbosa (Antioquia; 6°40' 54.7''N, 75°41' 10.4''W), Oporapa (Huila; 2°01'40.5''N, 75°59'43''W), and Ibagué (Tolima; 4°32'22.3"N, 75°05'37.1"W). Spiders were collected in July (Barbosa), August (Oporapa), and September (Ibague) 2019. Locations were selected based on the previous distribution reports and accessibility for the species in Colombia (Estrada‐Gomez et al., 2015; Valenzuela‐Rojas et al., 2019, 2020).

Adult individuals were collected during three consecutive nights between 20:00 and 04:00 hr by randomly following cardinal transects of 500m. However, most collection was done in what we found to be the “peak hours” of activity of the species (between 00.00 – 03.00 hr; Juan Carlos V‐R and Julio César G‐G, Personal Observation). For each individual, elevation, temperature, relative humidity, and mass (g) were recorded (Table S1). At each locality, in addition to the 20 specimens, three more individuals were taken for standardization of the DNA metabarcoding technique. After being euthanized by freezing, the collected individuals were stored in 96% alcohol in separate Falcon centrifuge tubes and transported to the Biology Laboratory of the University of Ibagué (Ibagué, Colombia). Subsequently, 96% alcohol washes were performed to remove impurities. Later, the distal parts of the legs (tarsus and metatarsus) were removed, since they do not contain gut diverticula (Macías‐Hernández et al., 2018). In all cases, dissection procedures were conducted using forceps and scissors, flame‐sterilized after each dissection to prevent cross‐contamination. We performed all laboratory activities on a clean and sterilized laboratory bench.

### 2.2 Preliminary assays and sample processing

The development of the blocking primers was carried out following the protocol established by Lafage et al. (2020). Mitochondrial cytochrome c oxidase subunit 1 (COI) sequences for 230 entries of Ctenidae spiders were downloaded from BOLD database (https://www.boldsystems.org/) and clustered using the “PrimerMiner” package v0.18 (Elbrecht & Leese, 2017). Sequences were aligned in Geneious 8.1.7 (Kearse et al., 2012) using MAFFT v7.017 (Katoh et al., 2002). PrimerMiner's “selectivetrim” function was used to dgHCO and mlCOIntF (Leray et al., 2013) binding sites and the alignment for each group was visualized with PrimerMiner to visually identify suitable primer binding sites. Sites conserved among target spider prey taxa (Hexapoda) but differing in *Phoneutria* sequences were selected. The sequence designs of the blocking primers were as follows:

noSPI 5′TACACGACGCTCTTCCGATCTTCATTTYCCHCGWATAAAYAAYATAAG3′ and dgHCO1 5′CAGACGTGTGCTCTTCCGATCAGGAGTAAACTTCAGGGTGACCAAARAAYCA3′.

Since dissection of the highly diverticulated gut is difficult, we performed three preliminary assays to check which portion of the body would contain the greatest proportion of prey DNA (prosoma, prosoma + opisthosoma, or the entire individual except tarsus and metatarsus). For each assay, three spiders—one from each sampling location—were used. Each sample was individually homogenized and DNA was extracted following standard protocols: DNA extraction of tissues was performed using the Qiagen DNEasy Tissue kit (Qiagen) under manufacturer's conditions. PCRs were carried out in 25 µl reaction volumes containing 2 µl of DNA extract with equal DNA concentrations (39 ng/µl), 12.5 µl of MyTaq mastermix (Biolone), and 2.5 µM of each primer (dgHCO and mlCOIntF primers (Leray et al., 2013) to amplify the COI region. Four different annealing temperatures were tested (40, 40.3, 40.9, and 48°C) in the preliminary assays which were previously shown to work well for *COI* (Lafage et al., 2020). The optimum temperature being determined as 48°C (Figure S1). Thermocycler conditions were as follows: initial denaturation at 95°C for 15 min; 30 cycles of 30 s at 94°C, 90 s at 48°C and 90 s at 72°C; and a final extension for 10 min at 72°C. Positive amplifications were confirmed by visual inspection of PCR products in 2% agarose gels. PCR products were purified using ExoSAP‐IT™ PCR Product Cleanup Reagent (Thermo Fisher Scientific). DNA concentration of the cleaned PCR products was determined using a Qubit fluorometer (Thermo Fisher). Purified PCR products (positive samples with dgHCO/mlCOIntF primers) were then Sanger‐sequenced and the resulting sequences processed using the sangeranalyse R package (v. 0.1) (https://github.com/roblanf/sangeranalyseR). After an initial PCR with Illumina‐adapted primers (Lafage et al., 2020), we performed a second PCR with Illumina Nextera Indices and defined DNA concentrations of amplicons using Qubit. Then, amplicons were pooled in equimolar volumes (100 ng each). Resulting libraries were sequenced on an Illumina MiSeq (v3 chemistry 2 × 300 bp cycle kit with 5% PhiX spike in) carried out by AIM (Advanced Identification Methods GmbH) following standard protocols (Kress & Erickson, 2007; Sang et al., 1997).

### 2.3 DNA metabarcoding diet analysis

Preliminary analyses with the blocking primers identified that the optimal annealing temperature was 48°C (Figure S1). We also found that the Prosoma + Opisthosoma region contained the highest relative abundance of prey sequences of the three body regions tested. We used these conditions for the processing of the 60 *P. boliviensis* samples from the three sampled locations for metabarcoding.

Based on the results of our preliminary assay, we extracted DNA from the prosoma and opisthosoma of each specimen. The metabarcoding of the *P. boliviensis* samples was performed independently for each of the 60 samples of the study with the Illumina platform using the noSPI/dgHCO1 blocking primers designed during the preliminary assays. The raw data from the sequencing via Illumina were processed firstly merging paired‐end reads, this step was made with ‐fastq_mergepairs (default settings), and then, cutadapt 1.18 was used to remove tags and primers with default settings (Kechin et al., 2017) using Python 2.7.15 to obtain the filtered reads. Sequences with a length of less than 300pb were eliminated using FastQC version 0.11.8 and VSEARCH 2.9.1 (de Sena Brandine & Smith, 2019). In addition, singleton and chimera sequences were filtered using VSEARCH 2.9.1 (Rognes et al., 2016) at maximum expected error = 1, to generate the final FASTQ files by sample, following the protocols proposed by Leidenfrost et al. (2020) and Liu et al. (2020) (Leidenfrost et al., 2020; Liu et al., 2020). The VSEARCH 2.9.1 program was used to dereplicate, clustering and assign the sequences to operative taxonomic units (OTUs) with 98% identity as the threshold in FASTA files. All sequences were then matched against the OTUs to create a consensus OTU table using usearch_global. Of a total of 2.410.269 initial reads, after quality filtering 358.054 pair‐end reads were obtained.

Sequences were blasted against the complete sequence database of the Barcode of Life Data systems (BOLD) in order to find the closest matches using the BOLD Identification Engine (http://www.boldsystems.org) (Ratnasingham & Hebert, 2007). Taxon nomenclature follows the catalogue used in the BOLD and NCBI databases (accessed on March 2020). When conflicting taxonomic assignments appeared in the database, we took the lowest nonconflicting taxonomic level indicated by the BOLD search (Federhen, 2012). Based on the FASTQ files for each individual, 256 OTUs were identified.

Based on these OTUs, different filters were applied according to standard exclusion criteria for this technique (Deagle et al., 2019; Lafage et al., 2020). Sequences with the following characteristics were eliminated: (a) all reads representing fewer than 0.01% of the total number of reads per sample, (b) sequences that corresponded to environmental DNA or intestinal microbiota, and (c) OTUs matching prey for genus/family level data through a search in both BOLD and from GenBank, NCBI databases that did not correspond to the geographical distribution of *P. boliviensis*. (d) Once these filters were applied, the OTUs with identity at ≥97% to those sequences were identified at the taxonomic level of species, those with ≥95% at the genus level, those with ≥90% at the family level, and sequences with ≥75% at the order level. Additionally, BINs (Barcode Index Numbers) were used to identify sequence clusters within the database, correlating with species in 98% of all cases (Lafage et al., 2020). After OTU filtering, a total of 105.583 sequences were retained, corresponding to 96 OTUs. In order to not underestimate the total reads, the filters were applied for each sample (percentage of reads per sample). Number of reads in sixty individuals is summarized in Table S3.

### 2.4 Data analyses

To test differences in body mass between the sexes and populations, the mass of males and females (g) was initially compared using the Wilcoxon–Mann–Whitney test with the package “coin,” and the mass between populations using the Kruskal–Wallis test with the package “stats” in R version 4.0.2. In addition, a Wilcoxon post hoc analysis with Bonferroni adjustment was used (R Development Core, 2018), and the mass of the different spiders was correlated with the estimated size of the different potential prey obtained using the bugGuide database (Bartlett, 2003) (Table S1). The data on the diet of *Phoneutria* were analyzed separately by sex and population. To compare the number of reads between sexes and populations, a chi‐square goodness‐of‐fit test was applied with Minitab 17 (Arend, 2010). Additionally, the confidence intervals between sexes under the binomial distribution were corroborated with Statpages (https://statpages.info). Bipartite prey–spider reads interaction network diagrams by sex and populations were created with Sankeymatic (http://sankeymatic.com/), accessed on March 2020.

We also compared the diet referred to as species richness of prey (i.e., alpha diversity over OTUs after applying all filtering procedures for the reads) detected for each of the three Colombian populations of *P. boliviensis* using individual‐based rarefaction curves (Hsieh et al., 2016) by calculating the Hill's numbers by means the q parameter (q0 = species richness) (Chao et al., 2014). The confidence intervals for the obtained curves were determined using the bootstrap method with 1,000 resampling replicates. All analyses and visualizations were performed in R version 3.5.2 (R Development Core, 2018), using the iNEXT package (Hsieh et al., 2016).

[Correction added on 18 April 2021 after first publication: "sample‐based rarefaction curves" has been changed to "individual‐based rarefaction curves" in the preceding paragraph.]

## 3 RESULTS

### 3.1 Mass of spiders

We found that the females have a mass of 3.79 ± 0.21 g (mean ± standard error), with females weighing from 1.86 to 6.08 g, while males weigh 2.25 ± 0.17 g, with individuals weighing from 1.01 to 4.64 g. The difference in mass between the sexes was highly significant (*p* < 0.001) (Table S1 and Figure S2, section a). When analyzing the body mass by locality, also significant differences were found (*p* = 0.027). We found that spiders from the Ibagué sampling zone tend to have a larger mass (mean of 3.61 g) compared to those from the Oporapa and Barbosa populations (mean of 2.62 and 2.82 g, respectively). These results are summarized in Figure S2, section b. No correlation (*R* ^2^ = 0.003) was observed between fresh spider mass (g) and prey size (mm).

### 3.2 DNA metabarcoding analysis in *Phoneutria boliviensis*

Of the 60 samples analyzed, and after applying all the filters, 57 had at least one prey (for two Barbosa spiders and one Ibagué spider, no prey was detected). We identified 96 different OTUs belonging to 10 different taxonomic orders (9 invertebrate and 1 vertebrate). Arthropods contributed the largest portion of the diet (97.9% of prey DNA recovered). The taxonomic orders with the greatest number of species represented in the diet (77% of prey DNA recovered, where a prey record is understood as a group of unique sequences recovered from the entire dataset) included Diptera (23), Coleoptera (20), Lepidoptera (17), and Orthoptera (14). The less diversely represented orders were Phasmatodea (2), Hemiptera (2), Araneae (2), and Dermaptera (1). By frequency of occurrence, Diptera made up 23.9%, while Coleoptera made up 20.8%, Lepidoptera 17.7%, and Orthoptera 14.6%. Vertebrates (Squamata) represented a small portion of the diet, accounting for only 2% of the sequences obtained from gut content analysis.

**FIGURE 2:**
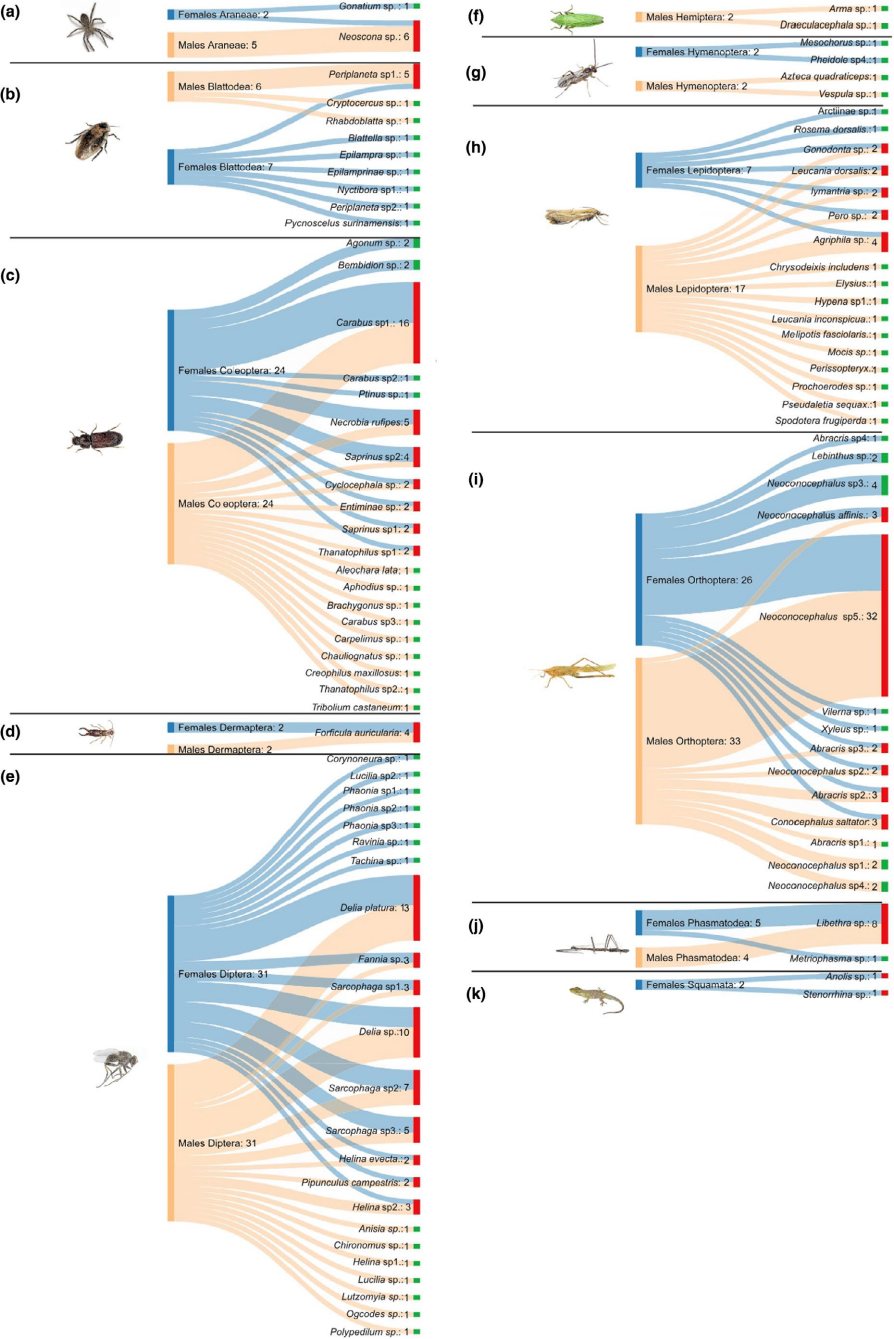
Intersex bipartite prey–spider species interaction network. Lines connect the males and females (left) to dietary species OTUs (bottom, colored by taxonomic order). The length of the boxes on the left reflects the number of prey analyzed for each OTU; the length of the boxes on the right reflects the relative abundance of each prey per OTU in each taxonomic order of prey in all samples in the dataset; and the width of the connecting lines reflects the relative reads abundance of each OTU within the diet of each taxonomic order. We show only the connections that represent ≥1% of the diet of each species (total *n* = 234 OTUs). (a) Araneae, (b) Blattodea, (c) Coleoptera, (d) Dermaptera, (e) Diptera, (f) Hemiptera, (g) Hymenoptera, (h) Lepidoptera, (i) Orthoptera, (j) Phasmatodea, (k) Squamata

The relative abundance analysis indicates that between 2 and a maximum of 64,287 reads were observed in each OTU. Of a total of 105,583 reads, 78.1% (82,537 reads) correspond to the order Orthoptera, followed by Diptera with 7.3% (7,776) and Blattodea with 6.4% (6,841). The remaining seven taxonomic orders represent only the remaining 8% of the reads in the diet of *P. boliviensis*. However, our results show a high dominance of reads for certain species. For example, the cricket of the genus *Neoconocephalus* (sp5) had the highest number of reads (64,287), which represented 60.8% of all reads (Table S2 and Figure 2).

A total of 234 prey records (number of individual prey items detected in each spider) were identified, belonging to 96 different taxa in the 57 spiders sampled (Table 1). The most abundant orders found as prey were again Diptera (62) and Orthoptera (59). Six OTUs belonging to the orthopteran genus *Neoconocephalus* were found, which were detected in 45 spiders, representing 19.2% of all registered prey items. For example, the OTU *Neoconocephalus* sp.5 is present in the diet of 32 of 57 spiders sampled. The prey taxa with the second highest frequency were the two species of flies belonging to the genus *Delia*, which had a frequency of 9.8% of all prey records.

**TABLE 1:** The diet of spider *Phonuetria boliviensis* from Colombia identified by DNA metabarcoding. Number of species corresponds to those identified to species, genera, and family level by sex and populations. Empty cells indicate no data registered. [Correction added on 18 April 2021 after first publication: Table caption has been updated to indicate the meaning of empty cells.]

Order	Species	Female count	Male count	Total	Total sex	Barbosa count	Total	Oporapa count	Total	Ibague count	Total
Araneae	*Gonatium* sp.	1	0	1	7	0	2	0	1	1	4
	*Neoscona* sp.	1	5	6		2		1		3	
Blattodea	*Blattella* sp.	1	0	1	13	0	4	1	6	0	3
	*Cryptocercus* sp.	0	1	1		0		1		0	
	*Epilampra* sp.	1	0	1		1		0		0	
	*Epilamprinae* sp.	1	0	1		0		1		0	
	*Nyctibora* sp.	1	0	1		0		1		0	
	*Periplaneta* sp1	1	4	5		2		1		2	
	*Periplaneta* sp2	1	0	1		0		0		1	
	*Pycnoscelus surinamensis*	1	0	1		1		0		0	
	*Rhabdoblatta* sp.	0	1	1		0		1		0	
Coleoptera	*Agonum* sp.	2	0	2	48	1	11	1	24	0	13
	*Aleochara lata*	0	1	1		0		1		0	
	*Aphodius* sp.	0	1	1		0		1		0	
	*Bembidion* sp.	2	0	2		0		2		0	
	*Brachygonus* sp.	0	1	1		0		0		1	
	*Carabus* sp1	8	8	16		3		8		5	
	*Carabus* sp2	1	0	1		0		1		0	
	*Carabus* sp3	0	1	1		0		0		1	
	*Carpelimus* sp.	0	1	1		1		0		0	
	*Chauliognathus* sp.	0	1	1		0		1		0	
	*Creophilus maxillosus*	0	1	1		0		0		1	
	*Cyclocephala* sp.	1	1	2		1		0		1	
	Entiminae sp.	1	1	2		0		1		1	
	*Necrobia rufipes*	3	2	5		1		2		2	
	*Ptinus* sp.	1	0	1		0		1		0	
	*Saprinus* sp1	1	1	2		2		0		0	
	*Saprinus* sp2	3	1	4		1		2		1	
	*Thanatophilus* sp1	1	1	2		1		1		0	
	*Thanatophilus* sp2	0	1	1		0		1		0	
	*Tribolium castaneum*	0	1	1		0		1		0	
Dermaptera	*Forficula auricularia*	2	2	4	4	0	0	4	4	0	0
Diptera	*Anisia* sp.	0	1	1	62	1	14	0	20	0	28
	*Chironomus* sp.	0	1	1		0		0		1	
	*Corynoneura* sp.	1	0	1		1		0		0	
	*Delia platura*	6	7	13		3		4		6	
	*Delia* sp.	4	6	10		4		2		4	
	*Fannia* sp.	2	1	3		0		2		1	
	*Helina* sp1	0	1	1		0		1		0	
	*Helina* sp2	1	2	3		0		2		1	
	*Helina evecta*	1	1	2		0		1		1	
	*Lucilia* sp1 (sp1)	0	1	1		0		1		0	
	*Lucilia* sp2	1	0	1		0		0		1	
	*Lutzomyia* sp	0	1	1		1		0		0	
	*Ogcodes* sp.	0	1	1		0		0		1	
	*Phaonia* sp1	1	0	1		0		1		0	
	*Phaonia* sp2	1	0	1		1		0		0	
	*Phaonia* sp3	1	0	1		0		1		0	
	*Pipunculus campestris*	1	1	2		0		1		1	
	*Polypedilum* sp.	0	1	1		0		0		1	
	*Ravinia* sp.	1	0	1		0		0		1	
	*Sarcophaga* sp1	2	1	3		1		0		2	
	*Sarcophaga* sp2	4	3	7		1		3		3	
	*Sarcophaga* sp3	3	2	5		1		1		3	
	*Tachina* sp.	1	0	1		0		0		1	
Hemiptera	*Arma* sp.	0	1	1	2	1	1	0	1	0	0
	*Draeculacephala* sp.	0	1	1		0		1		0	
Hymenoptera	*Azteca quadraticeps*	0	1	1	4	0	3	1	1	0	0
	*Mesochorus* sp.	1	0	1		1		0		0	
	*Pheidole* sp. (sp.)	1	0	1		1		0		0	
	*Vespula* sp.	0	1	1		1		0		0	
Lepidoptera	*Agriphila* sp.	1	3	4	24	1	10	3	8	0	6
	*Arctiinae* sp.	1	0	1		1		0		0	
	*Chrysodeixis includens*	0	1	1		1		0		0	
	*Elysius* sp.	0	1	1		0		1		0	
	*Gonodonta* sp.	1	1	2		1		0		1	
	*Hypena* sp.	0	1	1		0		1		0	
	*Leucania dorsalis*	1	1	2		1		0		1	
	*Leucania inconspicua*	0	1	1		0		1		0	
	*Lymantria* sp.	1	1	2		1		0		1	
	*Melipotis fasciolaris*	0	1	1		0		0		1	
	*Mocis* sp.	0	1	1		1		0		0	
	*Perissopteryx* sp.	0	1	1		1		0		0	
	*Pero* sp.	1	1	2		0		1		1	
	*Prochoerodes* sp.	0	1	1		1		0		0	
	*Pseudaletia sequax*	0	1	1		0		1		0	
	*Rosema dorsalis*	1	0	1		1		0		0	
	*Spodoptera frugiperda*	0	1	1		0		0		1	
Orthoptera	*Abracris* sp1	0	1	1	59	0	17	1	17	0	25
	*Abracris* sp2	1	2	3		0		2		1	
	*Abracris* sp3	1	1	2		0		1		1	
	*Abracris* sp4	1	0	1		0		0		1	
	*Conocephalus saltator*	1	2	3		1		2		0	
	*Lebinthus* sp.	2	0	2		0		0		2	
	*Neoconocephalus affinis*	2	1	3		0		2		1	
	*Neoconocephalus* sp1	0	2	2		2		0		0	
	*Neoconocephalus* sp2	1	1	2		1		0		1	
	*Neoconocephalus* sp3	4	0	4		4		0		0	
	*Neoconocephalus* sp4	0	2	2		0		0		2	
	*Neoconocephalus* sp5	11	21	32		9		9		14	
	*Vilerna* sp.	1	0	1		0		0		1	
	*Xyleus* sp.	1	0	1		0		0		1	
Phasmatodea	*Libethra* sp.	4	4	8	9	6	7	1	1	1	1
	*Metriophasma* sp.	1	0	1		1		0		0	
Squamata	*Anolis* sp.	1	0	1	2	1	1	0	0	0	1
	*Stenorrhina* sp.	1	0	1		0		0		1	
Total		108	126	234	234	70	70	83	83	81	81

### 3.3 Intersexual differences in prey composition of *Phoneutria boliviensis*

In this work, differences were identified in the consumption of prey between sexes of *P. boliviensis*. Of the 105,583 reads analyzed, 28.9% (30,516) correspond to prey detected in females and 71.1% (75,067) of the prey detected in males. For most orders, the number of reads of prey in males was more than double that of prey detected in females. Some taxonomic orders were observed only one sex, as in the case of Squamata in females or Hemiptera in males (Table S2). The prey–spider reads interaction network by sex is summarized in Figure S3, section a.

The greatest difference in the number of reads by prey between the sexes was found in the order Orthoptera. This order presents of 68.7% of reads in females compared with 82% in males. On the other hand, the results show us that of the total reads for this order (82,537), the females represent 25.4% (20,961), while the males represent 74.6% (61,576). When comparing the proportion of reads in the other taxonomic orders between the sexes, although they present significant differences between them (*p* = 0.0001), these are not as marked as those observed in Orthoptera.

A total of 234 prey records belonging to 96 different OTUs were observed, of which 108 were detected in females and 126 in males. The differences are mainly in the orders Orthoptera (females: 26 and males: 33), Lepidoptera (females: 7 and males: 17), Hemiptera (females: 0 and males: 2), and Squamata (females: 2 and males: 0). We identified 60 different prey species in females, while 65 different prey species were identified in males. Half (30) of the identified prey in the females are unique, while the other half (30) are shared by both sexes. Thirty‐five species were observed as unique in males. These results of the prey–spider species interaction network by sex are summarized in Figure S3.

### 3.4 Interpopulation differences in prey composition of *Phoneutria boliviensis*

**FIGURE 3:**
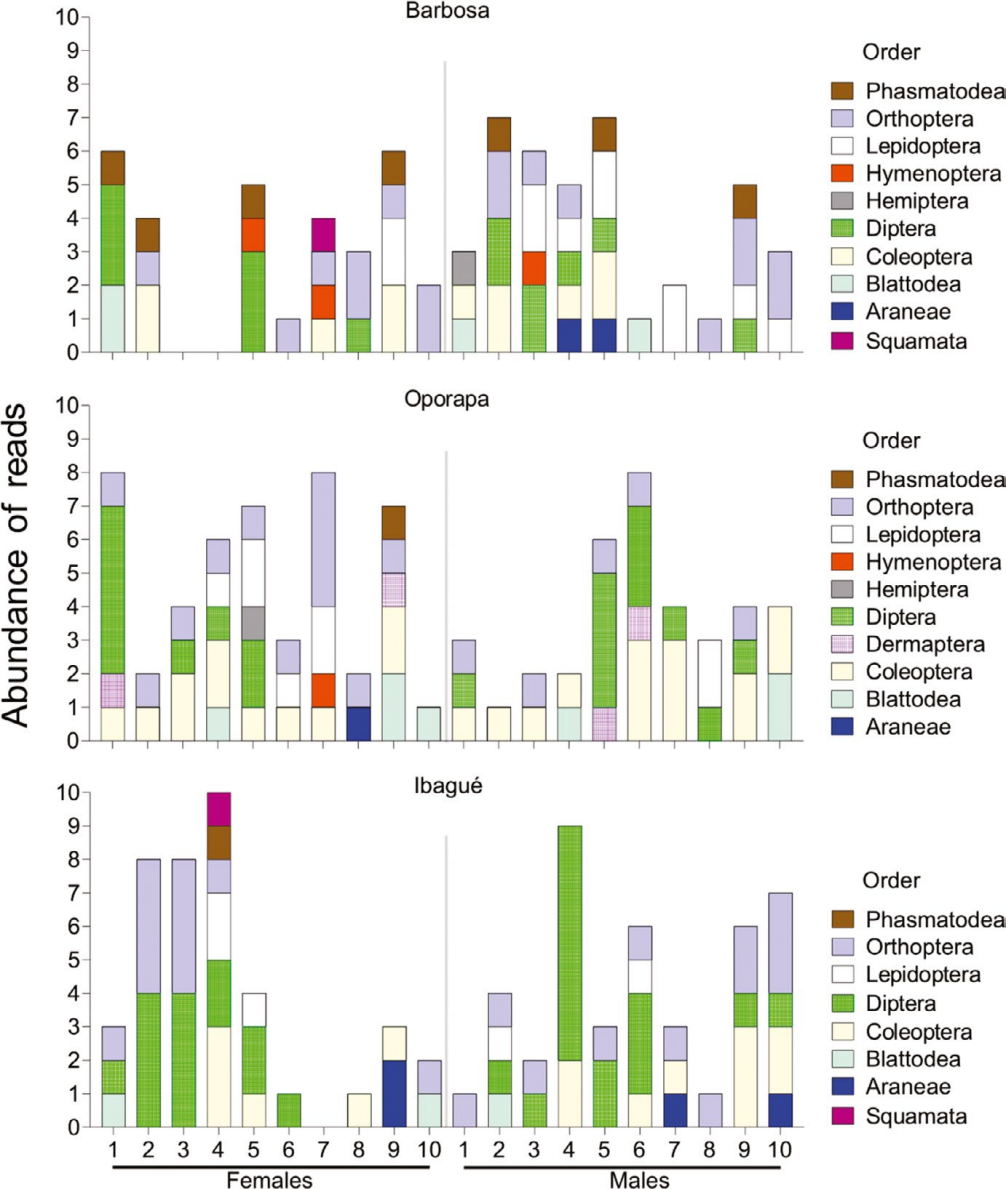
The relative abundance of reads per sample and locality. The boxes on the x‐axis represent each individual by population. The different colors represent the relative abundance of reads for each taxonomic order

Differences in the composition of diet in *P. boliviensis* were observed among the different populations. Of the 105,583 reads analyzed, 63.4% (66,970) correspond to prey detected in Barbosa, 13.1% (13,829) in Oporapa and 23.5% (24,784) of the prey detected in Ibagué (Table S2). The relative abundance of reads per sample and locality is shown in Figure 3.

**FIGURE 4:**
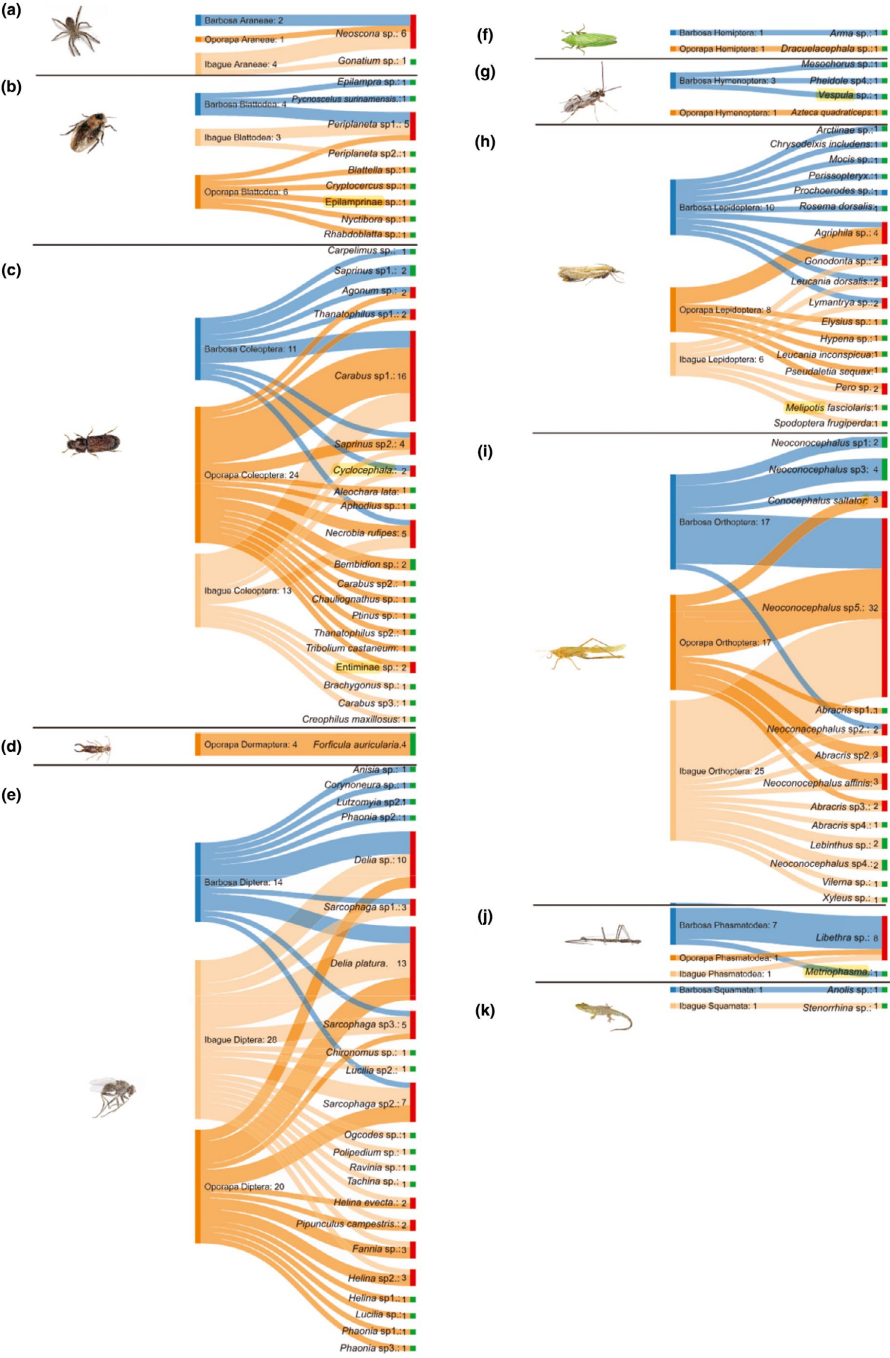
Interpopulation bipartite prey–spider species interaction network. Lines connect the Barbosa, Oporapa, and Ibagué localities (left) to dietary OTUs (bottom, colored by taxonomic order). The length of the boxes on the left reflects the number of preys analyzed for each OTU. The length of the boxes on the right reflects the relative abundance of each prey per OTU in each taxonomic order of prey in all samples in the dataset, and the width of the connecting lines reflects the relative reads abundance of each OTU within the diet of each taxonomic order. We show only the connections that represent ≥1% of the diet of each species (total *n* = 234 OTUs). (a) Araneae, (b) Blattodea, (c) Coleoptera, (d) Dermaptera, (e) Diptera, (f) Hemiptera, (g) Hymenoptera, (h) Lepidoptera, (i) Orthoptera, (j) Phasmatodea, (k) Squamata [Correction added on 18 April 2021 after first publication: the images of Figures 4 and 5 were previously interchanged and have now been corrected in this version.]

The greatest difference between populations in reads per prey is observed in the order Orthoptera. For example, with respect to the total reads for each population, this order presented 90.3% in Barbosa, 46.9% in Oporapa, and 62.5% in Ibagué. On the other hand, the results showed that of the total reads for this order (82,537), the population of Barbosa represents 73.4% (60,537), while Oporapa presented 7.8% (6,487) and Ibagué 18.8%. (15,513). Chi‐square goodness‐of‐fit test shows significant differences between reads of populations (*p* = 0.0001). The prey–spider reads interaction network by population is summarized in Figure 4.

**FIGURE 5:**
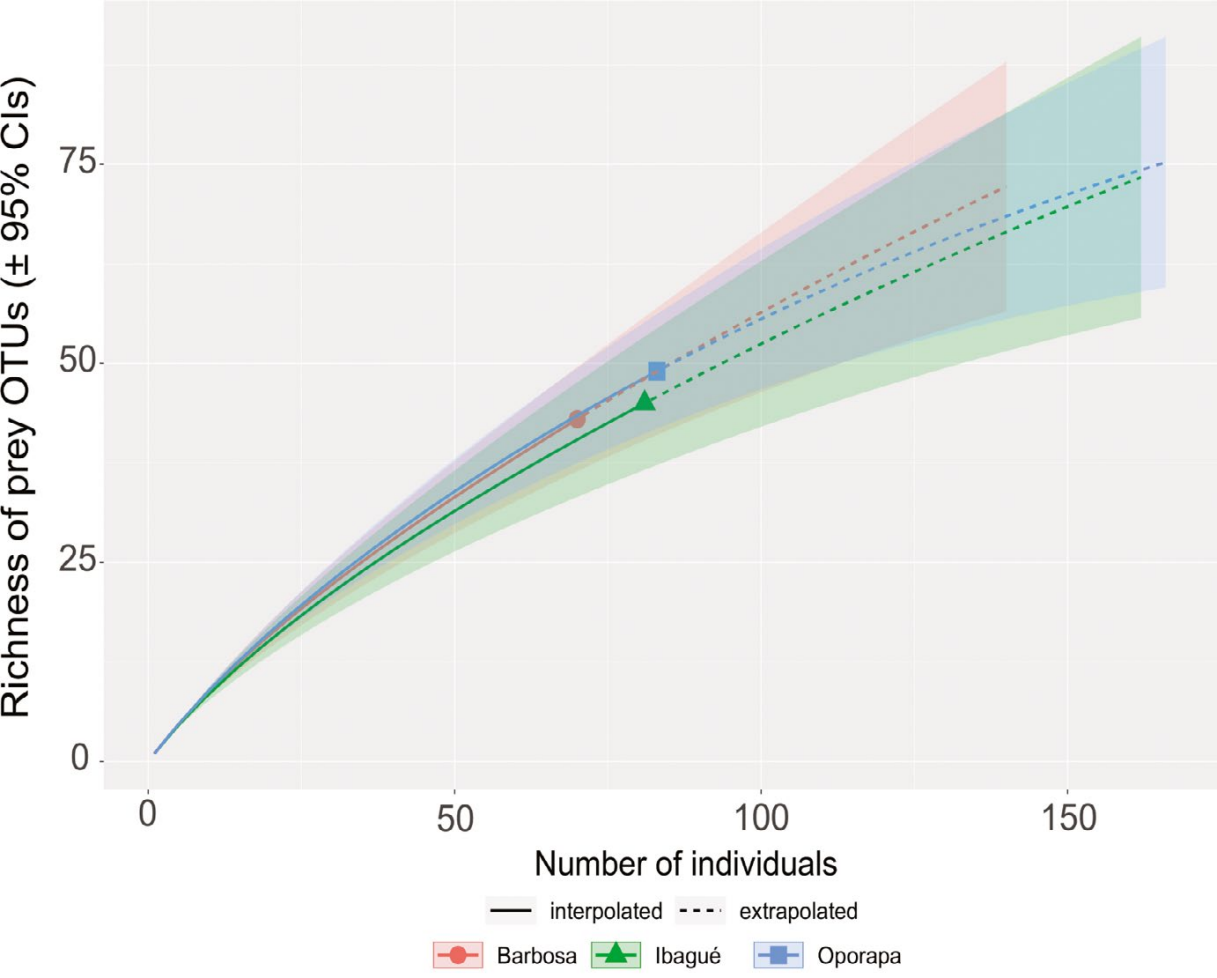
Individual‐based rarefaction curves of prey richness (q0) in the three Colombian populations of *P. boliviensis*, denoted by the three colors. Line types indicate whether estimates are interpolated (solid) or extrapolated (dashed). Ribbons indicate the 95% confidence intervals (CIs) obtained by the bootstrap method based on 1,000 replications [Correction added on 18 April 2021 after first publication: the images of Figures 4 and 5 were previously interchanged and have now been corrected in this version. Additionally, "Sample‐based rarefaction curves" has been corrected to 'Individual‐based rarefaction curves' in the Figure 5 caption]

A total of 234 prey belonging to 96 different OTUs were observed, of which 70 were detected in Barbosa, 83 in Oporapa, and 81 in Ibagué. In terms of prey richness, Barbosa had the highest number of prey OTUs across individuals, followed by Oporapa and Ibagué, which was supported by the rarefaction curves (Figure 5). The differences were mainly related to the orders Diptera (*n* = 14, 20, and 28, respectively), Orthoptera (*n* = 17, 17, and 25), and Coleoptera (*n* = 11, 24, and 13). In Barbosa, 35.7% (*n* = 25) of the species identified as prey are unique, while in Oporapa it has 30.1% (*n* = 25) and Ibagué with 23.5% (*n* = 19). Our results show that 21 to 26 spider prey species are shared between two or three spider populations.

[Correction added on 18 April 2021 after first publication: The sentence "The results of the prey‐spider species interaction network by population are summarized in Figure 4" has been removed from the end of the preceding paragraph.]

## 4 DISCUSSION

### 4.1 Diet of *Phoneutria boliviensis*

In trophic ecology studies, it is not always possible to track trophic links between predators and prey by direct observation. This is especially critical when observing small, wandering, or elusive animals with nocturnal or cryptic food‐web ecology (Sheppard & Harwood, 2005). Most studies based on molecular gut content analyses have focused on small‐sized spider families such as the Linyphiidae (Agustí et al., 2003; Macías‐Hernández et al., 2018), Lycosidae (Eitzinger et al., 2019; Lafage et al., 2020; Zhong et al., 2019), Theridiidae and Salticidae (Furlong et al., 2014; Whitaker et al., 2019), Tetragnathidae (Chapman et al., 2010; Toju & Baba, 2018), and Oxyopidae (Greenstone et al., 2014). In these studies, the procedure of extraction was either homogenizing the whole spider or crushing the abdomen, taking into account the amount of sample and the size of the spider. In our own study, the performance of the primer pair noSPI/dgHCO1 was compared in different body section samples of the spider, with the Prosoma + Opisthosoma sample showing the highest content of prey in *P. boliviensis*. Similar results were found in previous studies of other spider species, showing that this sampling of the body generally results in the largest prey content (Lafage et al., 2020; Macías‐Hernández et al., 2018). However, due to the use of blocking primers in our study, ctenid prey that were previously reported as prey for *Phoneutria*, such as *Spinoctenus*, *Ctenus,* and *Phoneutria* are blocked (Valenzuela‐Rojas et al., 2020). This might explain the low number of spiders we found as prey in our analyses.

In a recent study of gut content using DNA metabarcoding of four spider species of the genus *Tetragnatha*, Diptera, Hemiptera, and Lepidoptera were observed to be the most frequent in the diet of these species in order of importance (Kennedy et al., 2019). These results are strongly related with their capture strategy, since the genus *Tetragnatha* builds webs to obtain its food. However, this kind of spider is relatively small and web‐building and therefore difficult to compare to the large, wandering *P. boliviensis*.

In this work, we determined a wide diversity of potential prey (up to 10 orders and 96 species) consumed by *P. boliviensis* through DNA metabarcoding analysis of gut contents; identifying prey belonging to the orders Diptera, Coleoptera, Lepidoptera, and Orthoptera, principally. Our results show that *P. boliviensis* can consume, in addition to invertebrates, vertebrate species such as *Anolis* sp. (lizard) and *Stenorrhina* sp. (snake). Some vertebrates have previously been reported as part of the *P. boliviensis* diet (Valenzuela‐Rojas et al., 2019, 2020; Viera & Gonzaga, 2017).

Of the 57 spiders with gut contents identified, 52.6% (30) of them has at least one Diptera species as prey. According to our results, frequent consumption of some Diptera species could be considered as the basis of the diet of this spider. However, the study by Valenzuela et al. (Valenzuela‐Rojas et al., 2020) reported no captures of Diptera, hypothesizing that *P. boliviensis* has a preference for larger prey. They identified prey up to three times larger than the size of the spider itself, which is contrary to our observations of a high preference for Diptera prey, which are small species between 4 to 13 mm long (Bartlett, 2003).

Despite all the advantages of prey detection using DNA metabarcoding, and the large number of arthropod species with DNA barcodes (246,069) in the Barcode of Life Data Systems (BOLD) (Ratnasingham & Hebert, 2007), the low representativity of sequences from South America (Colombia with only 39.741 of 9.265.546 Specimen Records, accessed June 2020) reduces the likelihood that sequences are correctly assigned, which may lead to not identifying sequences of 77 species (identified only at the order level) of prey that were in the gut contents of the spider.

According to recent studies, DNA metabarcoding is mostly used to detect the presence of species in samples rather than their relative abundance, although sequence frequency is sometimes used as a proxy of species abundance (Aizpurua et al., 2018; Deagle et al., 2019). Several studies have demonstrated positive relationships between input species biomass and output sequencing reads (Krehenwinkel et al., 2017). Therefore, based on our analyses of relative abundance of reads of prey, the most abundant taxonomic groups were Orthoptera, Diptera, and Blattodea, respectively (see Table S2 and Figure S3), which could mean that the highest biomass associated with stomach content in *P. boliviensis* would be associated mainly with the cricket of the genus *Neoconocephalus* and the species of Diptera detected as prey. On the other hand, according to Sint et al. (Sint et al., 2011, 2015), the molecular detection of prey in spiders has a high sensitivity up to 84‐hr postfeeding; which would explain both the high number of prey per spider detected in this work (234), and the variation in the reads of the same prey species (i.e., the cricket of the genus *Neoconocephalus*, detected in 37/57 spider’ guts).

### 4.2 Differences between males and females

In most animals, males and females show marked differences in primary and secondary sexual traits. The sexual dimorphism literature pertaining to invertebrates is fragmented, particularly for arachnids (McLean et al., 2018). Spiders are sexually dimorphic in various morphological, behavioral, and life‐history traits (Cordellier et al., 2020). Our study provides evidence for both sexual dimorphism (significant differences in mass between females and males), and a different trophic ecology in males and females of *P. boliviensis*. We identified that the males on average are smaller than the females; however, we found that some males are larger than some females. Adult male and female spiders generally differ in body weight, but they can be of similar size and shape or differ markedly, depending on species (Cordellier et al., 2020). These analyses would suggest that a differential trophic environment could affect this character (see above for our analysis by location).

We found sexual significant differences in diet in our analysis of gut contents of *P. boliviensis*. Our results show that of the total reads, 28.9% are present in females and 71.1% detected in males. The larger number of prey reads in males versus females is surprising given that females tend to be larger, and often males stop or greatly reduce their rate of feeding when sexually mature. In a study on wolf spiders, differences in predatory behavior were observed between males and females (Persons & Uetz, 1999). In this study, it was observed that male wolf spiders travel greater distances, with greater hunting efforts compared to females. Due to this, these results would be supported since the higher number of prey reads in males would suggest a greater hunting effort (similar to male wolf spiders) and a greater acceptance of different prey in comparison with the females in *P. boliviensis*.

Our results show that wild female *P. boliviensis* prey on vertebrates. Different studies under laboratory conditions have shown the tendency of *P. boliviensis* to be euryphagus given its great voracity, as well as its capacity to consume small vertebrates (Valenzuela‐Rojas et al., 2019). Through the analysis of gut contents by metabarcoding, these findings are corroborated in the wild.

The difference in the number of intakes of prey or variety of food items is usually attributed to differences in size, either total size or specific structures such as chelicerae or carapace (Foellmer & Moya‐Larano, 2007). Body size dimorphism may be the result of selection for many factors, such as reproductive success, hyperpredation, or dispersal capacity among others (Crawley, 2009). In our results, males are smaller than females and also have a greater number of prey items than females, which supports the hypothesis that in spiders with sexual dimorphism a larger size does not necessarily imply that a greater range of prey can be captured (McLean et al., 2018). Similarly, the capacity to consume certain prey is influenced by multiple factors, not only morphological differences between sexes, but also differential predatory behavior and the active search for females by males (Kotiaho et al., 1998). The toxicity of *P. boliviensis* venom to beetles is higher in males than in females, which could ensure greater success in hunting, without ruling out the possibility that it is used defensively (Valenzuela‐Rojas et al., 2019).

### 4.3 Differences between populations

Although the number of prey items among the Barbosa, Oporapa, and Ibagué populations is very similar (70, 83, and 81, respectively), differences exist in the type of prey. When comparing the diet in *P. boliviensis* among populations, the diversity (number of species) of Coleoptera was higher in Oporapa, representing twice as many items as the other two populations studied. Furthermore, it was the only population where the order Dermaptera was consumed, while in Ibagué the most consumed order was Diptera followed by Orthoptera. Finally, Barbosa was the locality with the lowest number of prey species. The three localities present differences in the prey species that compose the diet, which may indicate that the selection of prey species may be in good part be determined by their availability (Klaus Birkhofer & Wolters, 2012; Eitzinger et al., 2019; Hambäck et al., 2016). Nevertheless, the analysis of relative read counts by population shows that spiders from Barbosa have two or three times more reads than the populations of Oporapa and Ibagué. One hypothesis that could explain this behavior would be that larger spiders have a greater prey capacity. However, our results do not identify a clear correlation (*R* ^2^ = 0.003) between read count per population and the body size of the spider (in terms of fresh mass).

The proportions of each order of prey show marked differences between populations, especially in the order Orthoptera. These differences between populations may be influenced by the availability of prey in each population, and these in turn are influenced by biogeographical characteristics such as the Andes mountain range that crosses the populations and could affect the ranges of certain prey species, as well as factors such as temperature, humidity, and anthropogenic pressures (Ramirez‐Villegas et al., 2014). According to the environmental data of relative humidity, temperature, and altitude, some differences are observed between the populations (see Table S1), which would justify the differences between them at the trophic level.

## 5 CONCLUSIONS

This project contributed to the knowledge of the trophic ecology of *P. boliviensis* using the DNA metabarcoding approach, confirming its euryphagous feeding behavior. We show a wider range of prey species than reported previously for this species in Colombia. Some prey species are reported here for the first time. Our results also could be the first evidence that males of *P. boliviensis* apparently have a greater hunting effort, as indicated by an increased number of prey and reads compared to females. This suggests different predatory strategies between the sexes, perhaps based on different energy requirements. Similarly, there is a small difference in trophic ecology depending on the locality (comparing our records of diet composition and taxa richness), again confirming the generalist nature and flexibility of the diet. However, our results differ from previous findings using different diet assessment techniques in the field and the laboratory (Valenzuela‐Rojas et al., 2019, 2020). Particularly the high fraction of Diptera in the diet, we found could not have been anticipated based on these previous findings and requires further corroboration. In addition, it is necessary to extend the studies with DNA barcoding in South America, in order to identify, at the species level, the possible prey and the ecological impact of this predatory spider.

## CONFLICT OF INTEREST

All authors declare no conflict of interest including any financial, personal, or other relationships with other people or organizations.

## AUTHOR CONTRIBUTIONS

**Diego Sierra Ramirez** involved in methodology (equal), spiders collecting (equal), mass measures of the spiders (lead), data processing (lead), statistical processing(equal), construction, and revision of the manuscript (equal). **Giovany Guevara** involved in conceptualization (equal), methodology (equal), execution of diversity analysis (lead), statistical analysis (equal), and review of the manuscript (equal). **Lida Marcela Franco Pérez** involved in conceptualization (equal), methodology (equal), construction (equal), and review of the manuscript (equal). **Arie van der Meijden** involved in conceptualization and review of the draft (lead). **Julio César González‐Gómez** involved in spiders collecting (supporting), mass measures of the spiders (supporting), review (equal), and comments on the draft (equal), **Juan Carlos Valenzuela‐Rojas** involved in spiders collecting (lead), review, and comments on the draft (supporting). **Carlos Fernando Prada Quiroga** involved in conceptualization (equal) methodology (lead), data processing (equal), construction, and revision of the manuscript (equal).

## Supporting information

Fig S1

Fig S2

Fig S3

Table S1

Table S2

Table S3

## Data Availability

The data that support the findings of this study are available on dyad ID https://doi.org/10.5061/dryad.x3ffbg7hm https://datadryad.org/stash/share/XGlI3pp1xVCfmCKmzcFEVxyBWDZ4f6xJiIk3UOFVyYw.
