# Supplementary material for: Deciphering the diet of a wandering spider (Phoneutria boliviensis; Araneae: Ctenidae) by DNA metabarcoding of gut contents
Source: Ecol Evol. 2021 Mar 6;11(11):5950–65. doi: 10.1002/ece3.7320 (PMC8207164; doi:10.1002/ece3.7320)
Supplement: Supplementary file 4 — Table S1 [file ECE3-11-5950-s006.docx]

Supplementary Table S1. Elevation, temperature, relative humidity and mass data in the sampled localities.

| ID | Population | Sex | Elevation | Temperature (C°) | RH | Mass (g) |
| --- | --- | --- | --- | --- | --- | --- |
| 1HB | Barbosa - Antioquia | Female | 1380 | 24,5 | 56,5 | 4,57 |
| 2HB | Barbosa - Antioquia | Female | 1380 | 24,5 | 56,5 | 3,15 |
| 3HB | Barbosa - Antioquia | Female | 1380 | 24,5 | 56,5 | 3,6 |
| 4HB | Barbosa - Antioquia | Female | 1380 | 24,5 | 56,5 | 2,2 |
| 5HB | Barbosa - Antioquia | Female | 1380 | 24,5 | 56,5 | 3,0 |
| 6HB | Barbosa - Antioquia | Female | 1349 | 25,9 | 55,5 | 2,2 |
| 7HB | Barbosa - Antioquia | Female | 1349 | 25,9 | 55,5 | 5,9 |
| 8HB | Barbosa - Antioquia | Female | 1349 | 25,9 | 55,5 | 5,2 |
| 9HB | Barbosa - Antioquia | Female | 1349 | 25,9 | 55,5 | 4,1 |
| 10HB | Barbosa - Antioquia | Female | 1349 | 25,9 | 55,5 | 3,1 |
| 1MB | Barbosa - Antioquia | Male | 1380 | 24,5 | 56,5 | 1,47 |
| 2MB | Barbosa - Antioquia | Male | 1380 | 24,5 | 56,5 | 1,83 |
| 3MB | Barbosa - Antioquia | Male | 1349 | 25,9 | 55,5 | 2,1 |
| 4MB | Barbosa - Antioquia | Male | 1349 | 25,9 | 55,5 | 2,2 |
| 5MB | Barbosa - Antioquia | Male | 1349 | 25,9 | 55,5 | 1,9 |
| 6MB | Barbosa - Antioquia | Male | 1399 | 24,8 | 68,5 | 1,8 |
| 7MB | Barbosa - Antioquia | Male | 1399 | 24,8 | 68,5 | 1,76 |
| 8MB | Barbosa - Antioquia | Male | 1399 | 24,8 | 68,5 | 2,11 |
| 9MB | Barbosa - Antioquia | Male | 1399 | 24,8 | 68,5 | 2,64 |
| 10MB | Barbosa - Antioquia | Male | 1399 | 22,2 | 66,8 | 1,6 |
| 1HO | Oporapa - Huila | Female | 1233 | 22,5 | 76,9 | 1,99 |
| 2HO | Oporapa - Huila | Female | 1233 | 22,5 | 76,9 | 3,36 |
| 3HO | Oporapa - Huila | Female | 1222 | 20 | 82,1 | 4,14 |
| 4HO | Oporapa - Huila | Female | 1222 | 20 | 82,1 | 2,73 |
| 5HO | Oporapa - Huila | Female | 1222 | 19,5 | 88,5 | 4,87 |
| 6HO | Oporapa - Huila | Female | 1222 | 19,5 | 88,5 | 2,65 |
| 7HO | Oporapa - Huila | Female | 1222 | 19,5 | 88,5 | 6,08 |
| 8HO | Oporapa - Huila | Female | 1222 | 19,5 | 88,5 | 1,86 |
| 9HO | Oporapa - Huila | Female | 1222 | 19,5 | 88,5 | 3,44 |
| 10HO | Oporapa - Huila | Female | 1222 | 19,5 | 88,5 | 3,94 |
| 1MO | Oporapa - Huila | Male | 1152 | 20,7 | 75,9 | 1,48 |
| 2MO | Oporapa - Huila | Male | 1233 | 22,5 | 76,9 | 1,01 |
| 3MO | Oporapa - Huila | Male | 1238 | 20 | 74,1 | 2,23 |
| 4MO | Oporapa - Huila | Male | 1222 | 20 | 82,1 | 1,42 |
| 5MO | Oporapa - Huila | Male | 1222 | 20 | 82,1 | 1,14 |
| 6MO | Oporapa - Huila | Male | 1222 | 20 | 82,1 | 2,02 |
| 7MO | Oporapa - Huila | Male | 1222 | 19,5 | 88,5 | 2,61 |
| 9MO | Oporapa - Huila | Male | 1389 | 26,5 | 70,6 | 2,66 |
| 10MO | Oporapa - Huila | Male | 1530 | 20,9 | 70,31 | 1,39 |
| 11MO | Oporapa - Huila | Male | 1530 | 20,9 | 70,31 | 1,15 |
| 1HI | Ibague - Tolima | Female | 663 | 28,2 | 74,1 | 3,45 |
| 2HI | Ibague - Tolima | Female | 663 | 28,2 | 74,1 | 3,74 |
| 3HI | Ibague - Tolima | Female | 663 | 28,2 | 74,1 | 3,45 |
| 4HI | Ibague - Tolima | Female | 641 | 27,1 | 68,1 | 4,18 |
| 5HI | Ibague - Tolima | Female | 641 | 27,1 | 68,1 | 4,38 |
| 6HI | Ibague - Tolima | Female | 641 | 27,1 | 68,1 | 4,98 |
| 8HI | Ibague - Tolima | Female | 646 | 27,8 | 69,6 | 5,11 |
| 9HI | Ibague - Tolima | Female | 646 | 27,8 | 69,6 | 4,8 |
| 10HI | Ibague - Tolima | Female | 655 | 27,7 | 70,4 | 2,44 |
| 11HI | Ibague - Tolima | Female | 655 | 27,7 | 70,4 | 5,11 |
| 1MI | Ibague - Tolima | Male | 663 | 28,2 | 74,1 | 2,96 |
| 2MI | Ibague - Tolima | Male | 641 | 27,1 | 68,1 | 2,03 |
| 3MI | Ibague - Tolima | Male | 641 | 27,1 | 68,1 | 1,68 |
| 4MI | Ibague - Tolima | Male | 655 | 27,7 | 70,4 | 2,0 |
| 5MI | Ibague - Tolima | Male | 655 | 27,7 | 70,4 | 4,34 |
| 6MI | Ibague - Tolima | Male | 655 | 27,7 | 70,4 | 4,48 |
| 7MI | Ibague - Tolima | Male | 655 | 27,7 | 70,4 | 1,81 |
| 8MI | Ibague - Tolima | Male | 655 | 27,7 | 70,4 | 2,77 |
| 9MI | Ibague - Tolima | Male | 655 | 27,7 | 70,4 | 3,94 |
| 10MI | Ibague - Tolima | Male | 655 | 27,7 | 70,4 | 4,64 |
