# Supplementary material for: Deciphering the diet of a wandering spider (Phoneutria boliviensis; Araneae: Ctenidae) by DNA metabarcoding of gut contents
Source: Ecol Evol. 2021 Mar 6;11(11):5950–65. doi: 10.1002/ece3.7320 (PMC8207164; doi:10.1002/ece3.7320)
Supplement: Supplementary file 5 — Table S2 [file ECE3-11-5950-s001.docx]

Supplementary Table S2. Diet of the *P. boliviensis* spider from Colombia identified by DNA metabarcoding. Number of reads at species and order levels

| Order | Specie | Female | Male |  | Total order | Barbosa | Total order | Oporapa | Total order | Ibague | Total order |
| --- | --- | --- | --- | --- | --- | --- | --- | --- | --- | --- | --- |
| Araneae | *Gonatium* ssp | 2 | 0 | 2 | 843 | 0 | 5 | 0 | 2 | 2 | 836 |
|  | *Neoscona* ssp. | 4 | 837 | 841 |  | 5 |  | 2 |  | 834 |  |
| Blattodea | *Blattella* ssp. | 37 | 0 | 37 | 6841 | 0 | 1354 | 37 | 678 | 0 | 4809 |
|  | *Cryptocercus* ssp. | 0 | 4 | 4 |  | 0 |  | 4 |  | 0 |  |
|  | *Epilampra* ssp. | 1295 | 0 | 1295 |  | 1295 |  | 0 |  | 0 |  |
|  | *Epilamprinae* ssp. | 69 | 0 | 69 |  | 0 |  | 69 |  | 0 |  |
|  | *Nyctibora* ssp. | 447 | 0 | 447 |  | 0 |  | 447 |  | 0 |  |
|  | *Periplaneta* ssp1 | 292 | 4532 | 4824 |  | 5 |  | 12 |  | 4807 |  |
|  | *Periplaneta* ssp2 | 2 | 0 | 2 |  | 0 |  | 0 |  | 2 |  |
|  | *Pycnoscelus surinamensis* | 54 | 4 | 58 |  | 54 |  | 4 |  | 0 |  |
|  | *Rhabdoblatta* ssp. | 0 | 105 | 105 |  | 0 |  | 105 |  | 0 |  |
| Coleoptera | *Agonum* ssp. | 14 | 0 | 14 | 3300 | 10 | 352 | 4 | 2065 | 0 | 883 |
|  | *Aleochara lata* | 6 | 0 | 6 |  | 0 |  | 6 |  | 0 |  |
|  | *Aphodius* ssp. | 0 | 10 | 10 |  | 0 |  | 10 |  | 0 |  |
|  | *Bembidion* ssp. | 12 | 0 | 12 |  | 0 |  | 12 |  | 0 |  |
|  | *Brachygonus* ssp. | 0 | 8 | 8 |  | 0 |  | 0 |  | 8 |  |
|  | *Carabus* ssp1. | 387 | 265 | 652 |  | 80 |  | 304 |  | 268 |  |
|  | *Carabus* ssp2. | 84 | 0 | 84 |  | 0 |  | 84 |  | 0 |  |
|  | *Carabus* ssp3. | 0 | 332 | 332 |  | 0 |  | 0 |  | 332 |  |
|  | *Carpelimus* ssp. | 0 | 22 | 22 |  | 22 |  | 0 |  | 0 |  |
|  | *Chauliognathus* ssp. | 0 | 229 | 229 |  | 0 |  | 229 |  | 0 |  |
|  | *Creophilus maxillosus* | 0 | 58 | 58 |  | 0 |  | 0 |  | 58 |  |
|  | *Cyclocephala* ssp. | 13 | 136 | 149 |  | 136 |  | 0 |  | 13 |  |
|  | *Entiminae* ssp. | 248 | 5 | 253 |  | 0 |  | 248 |  | 5 |  |
|  | *Necrobia rufipes* | 229 | 49 | 278 |  | 25 |  | 170 |  | 83 |  |
|  | *Ptinus* ssp. | 35 | 0 | 35 |  | 0 |  | 35 |  | 0 |  |
|  | *Saprinus* ssp1. | 34 | 9 | 43 |  | 43 |  | 0 |  | 0 |  |
|  | *Saprinus* ssp2. | 848 | 3 | 851 |  | 21 |  | 714 |  | 116 |  |
|  | *Thanatophilus* ssp1. | 2 | 15 | 17 |  | 15 |  | 2 |  | 0 |  |
|  | *Thanatophilus* ssp2. | 0 | 122 | 122 |  | 0 |  | 122 |  | 0 |  |
|  | *Tribolium castaneum* | 0 | 125 | 125 |  | 0 |  | 125 |  | 0 |  |
| Dermaptera | *Forficula auricularia* | 39 | 237 | 276 | 276 | 0 | 0 | 276 | 276 | 0 | 0 |
| Diptera | *Anisia* ssp. | 0 | 28 | 28 | 7776 | 28 | 2203 | 0 | 3040 | 0 | 2533 |
|  | *Chironomus* ssp. | 0 | 2 | 2 |  | 0 |  | 0 |  | 2 |  |
|  | *Corynoneura* ssp. | 9 | 0 | 9 |  | 9 |  | 0 |  | 0 |  |
|  | *Delia platura* | 1945 | 2469 | 4414 |  | 1059 |  | 1945 |  | 1410 |  |
|  | *Delia* ssp. | 374 | 1024 | 1398 |  | 643 |  | 277 |  | 478 |  |
|  | *Fannia* ssp | 97 | 2 | 99 |  | 0 |  | 35 |  | 64 |  |
|  | *Helina* ssp 1 | 0 | 7 | 7 |  | 0 |  | 7 |  | 0 |  |
|  | *Helina* ssp2 | 9 | 50 | 59 |  | 0 |  | 27 |  | 32 |  |
|  | *Helina evecta* | 23 | 3 | 26 |  | 0 |  | 23 |  | 3 |  |
|  | *Lucilia* ssp. | 0 | 44 | 44 |  | 0 |  | 44 |  | 0 |  |
|  | *Lucilia* ssp2. | 22 | 0 | 22 |  | 0 |  | 0 |  | 22 |  |
|  | *Lutzomyia* ssp. | 0 | 33 | 33 |  | 33 |  | 0 |  | 0 |  |
|  | *Ogcodes* ssp. | 0 | 34 | 34 |  | 0 |  | 0 |  | 34 |  |
|  | *Phaonia* ssp1. | 5 | 0 | 5 |  | 0 |  | 5 |  | 0 |  |
|  | *Phaonia* ssp2. | 26 | 0 | 26 |  | 26 |  | 0 |  | 0 |  |
|  | *Phaonia* ssp3. | 29 | 0 | 29 |  | 0 |  | 29 |  | 0 |  |
|  | *Pipunculus campestris* | 0 | 17 | 17 |  | 0 |  | 15 |  | 2 |  |
|  | *Polypedilum* ssp1. | 0 | 2 | 2 |  | 0 |  | 0 |  | 2 |  |
|  | *Ravinia* ssp1. | 27 | 0 | 27 |  | 0 |  | 0 |  | 27 |  |
|  | *Sarcophaga* ssp1. | 192 | 235 | 427 |  | 166 |  | 0 |  | 261 |  |
|  | *Sarcophaga* ssp2. | 548 | 202 | 750 |  | 16 |  | 610 |  | 124 |  |
|  | *Sarcophaga* ssp3. | 265 | 50 | 315 |  | 223 |  | 23 |  | 69 |  |
|  | *Tachina* ssp1. | 3 | 0 | 3 |  | 0 |  | 0 |  | 3 |  |
| Hemiptera | *Arma* ssp. | 0 | 263 | 263 | 425 | 263 | 263 | 0 | 162 | 0 | 0 |
|  | *Draeculacephala* ssp. | 0 | 162 | 162 |  | 0 |  | 162 |  | 0 |  |
| Hymenoptera | *Azteca quadraticeps* | 0 | 6 | 6 | 78 | 0 | 72 | 6 | 6 | 0 | 0 |
|  | *Mesochorus* ssp. | 10 | 0 | 10 |  | 10 |  | 0 |  | 0 |  |
|  | *Pheidole* ssp. | 60 | 0 | 60 |  | 60 |  | 0 |  | 0 |  |
|  | *Vespula* ssp. | 0 | 2 | 2 |  | 2 |  | 0 |  | 0 |  |
| Lepidoptea | *Spodoptera frugiperda* | 0 | 77 | 77 | 1773 | 0 | 499 | 0 | 1101 | 77 | 173 |
|  | *Agriphila* ssp. | 48 | 54 | 102 |  | 23 |  | 79 |  | 0 |  |
|  | *Arctiinae* ssp. | 10 | 0 | 10 |  | 10 |  | 0 |  | 0 |  |
|  | *Chrysodeixis includens* | 0 | 71 | 71 |  | 71 |  | 0 |  | 0 |  |
|  | *Elysius* ssp. | 0 | 34 | 34 |  | 0 |  | 34 |  | 0 |  |
|  | *Gonodonta* sp. | 5 | 30 | 35 |  | 30 |  | 0 |  | 5 |  |
|  | *Hypena* ssp. | 0 | 332 | 332 |  | 0 |  | 332 |  | 0 |  |
|  | *Leucania dorsalis* | 19 | 288 | 307 |  | 288 |  | 0 |  | 19 |  |
|  | *Leucania inconspicua* | 0 | 128 | 128 |  | 0 |  | 128 |  | 0 |  |
|  | *Lymantria* ssp. | 8 | 19 | 27 |  | 19 |  | 0 |  | 8 |  |
|  | *Melipotis fasciolaris* | 0 | 59 | 59 |  | 0 |  | 0 |  | 59 |  |
|  | *Mocis* ssp. | 0 | 26 | 26 |  | 26 |  | 0 |  | 0 |  |
|  | *Perissopteryx* ssp. | 0 | 7 | 7 |  | 7 |  | 0 |  | 0 |  |
|  | *Pero* ssp. | 522 | 5 | 527 |  | 0 |  | 522 |  | 5 |  |
|  | *Prochoerodes* ssp. | 0 | 9 | 9 |  | 9 |  | 0 |  | 0 |  |
|  | *Pseudaletia sequax* | 0 | 6 | 6 |  | 0 |  | 6 |  | 0 |  |
|  | *Rosema dorsalis* | 16 | 0 | 16 |  | 16 |  | 0 |  | 0 |  |
| Orthoptera | *Abracris* ssp1. | 0 | 41 | 41 | 82537 | 0 | 60537 | 41 | 6487 | 0 | 15513 |
|  | *Abracris* ssp2. | 1485 | 234 | 1719 |  | 0 |  | 234 |  | 1485 |  |
|  | *Abracris* ssp3. | 6 | 276 | 282 |  | 0 |  | 276 |  | 6 |  |
|  | *Abracris* ssp4. | 20 | 0 | 20 |  | 0 |  | 0 |  | 20 |  |
|  | *Conocephalus saltator* | 89 | 1495 | 1584 |  | 89 |  | 1495 |  | 0 |  |
|  | *Lebinthus* ssp1. | 83 | 0 | 83 |  | 0 |  | 0 |  | 83 |  |
|  | *Neoconocephalus* ssp2 | 2 | 4 | 6 |  | 4 |  | 0 |  | 2 |  |
|  | *Neoconocephalus affinis* | 304 | 3 | 307 |  | 0 |  | 304 |  | 3 |  |
|  | *Neoconocephalus* ssp1 | 0 | 23 | 23 |  | 23 |  | 0 |  | 0 |  |
|  | *Neoconocephalus* ssp3 | 14053 | 0 | 14053 |  | 14053 |  | 0 |  | 0 |  |
|  | *Neoconocephalus* ssp4 | 0 | 69 | 69 |  | 0 |  | 0 |  | 69 |  |
|  | *Neoconocephalus* ssp5 | 4856 | 59431 | 64287 |  | 46368 |  | 4137 |  | 13782 |  |
|  | *Vilerna* ssp. | 24 | 0 | 24 |  | 0 |  | 0 |  | 24 |  |
|  | *Xyleus* ssp. | 39 | 0 | 39 |  | 0 |  | 0 |  | 39 |  |
| Phasmatodea | *Libethra* ssp. | 290 | 604 | 894 | 969 | 847 | 922 | 12 | 12 | 35 | 35 |
|  | *Metriophasma* ssp. | 75 | 0 | 75 |  | 75 |  | 0 |  | 0 |  |
| Squamata | *Anolis* ssp. | 763 | 0 | 763 | 765 | 763 | 763 | 0 | 0 | 0 | 2 |
|  | *Stenorrhina* ssp. | 2 | 0 | 2 |  | 0 |  | 0 |  | 2 |  |
| Total |  | 30516 | 75067 | 105583 | 105583 | 66970 | 66970 | 13829 | 13829 | 24784 | 24784 |
