# Supplementary material for: Deciphering the diet of a wandering spider (Phoneutria boliviensis; Araneae: Ctenidae) by DNA metabarcoding of gut contents
Source: Ecol Evol. 2021 Mar 6;11(11):5950–65. doi: 10.1002/ece3.7320 (PMC8207164; doi:10.1002/ece3.7320)
Supplement: Supplementary file 6 — Table S3 [file ECE3-11-5950-s005.docx]

Supplementary Table S3. Number of reads in sixty individuals, 10 Barbosa females (F - B), 10 Barbosa males (M - B), 10 Ibague females (F - I), 10 Ibague males (M - I), 10 Oporapa females (F - O) and 10 Oporapa males (M - O) females (F - O) and 10 Oporapa males (M - O)

| ID F-B | N reads | ID M-B | N reads | ID F-I | N reads | ID M-I | N reads | ID F-O | N reads | ID M-O | N reads |
| --- | --- | --- | --- | --- | --- | --- | --- | --- | --- | --- | --- |
| 1FB | 1410 | 1MB | 285 | 1FI | 368 | 1MI | 19 | 1FO | 692 | 1MO | 2395 |
| 2FB | 440 | 2MB | 15966 | 2FI | 2089 | 2MI | 4610 | 2FO | 180 | 2MO | 40 |
| 1FB | 0 | 3MB | 2261 | 3FI | 1714 | 3MI | 5 | 3FO | 766 | 3MO | 39 |
| 2FB | 0 | 4MB | 13808 | 4FI | 1073 | 4MI | 642 | 4FO | 75 | 4MO | 314 |
| 1FB | 419 | 5MB | 923 | 5FI | 134 | 5MI | 1489 | 5FO | 2173 | 5MO | 1610 |
| 2FB | 7068 | 6MB | 2 | 6FI | 6 | 6MI | 9264 | 6FO | 315 | 6MO | 73 |
| 1FB | 5049 | 7MB | 30 | 8FI | 0 | 7MI | 831 | 7FO | 79 | 7MO | 1144 |
| 2FB | 887 | 8MB | 5 | 9FI | 103 | 8MI | 162 | 8FO | 599 | 9MO | 53 |
| 1FB | 2201 | 9MB | 856 | 10FI | 17 | 9MI | 607 | 9FO | 627 | 10MO | 1310 |
| 2FB | 681 | 10MB | 14679 | 11FI | 10 | 10MI | 1641 | 10FO | 1341 | 11MO | 4 |
